# Association between IgG responses against the nucleocapsid proteins of alphacoronaviruses and COVID-19 severity

**DOI:** 10.3389/fimmu.2022.889836

**Published:** 2022-09-07

**Authors:** Julius Nückel, Elisa Planatscher, Anne Wiebe Mohr, Karolin Deichl, Hrvoje Mijočević, Martin Feuerherd, Lisa Wolff, Johanna Erber, Jochen Schneider, Michael Quante, Christoph Winter, Jürgen Ruland, Alexander Hapfelmeier, Wolfgang Hammerschmidt, Andreas Moosmann, Ulrike Protzer, Uta Behrends, Josef Mautner

**Affiliations:** ^1^ Children’s Hospital, School of Medicine, Technische Universität München, Munich, Germany; ^2^ German Centre for Infection Research (DZIF) partner site Munich, Munich, Germany; ^3^ DZIF Research Group “Host Control of Viral Latency and Reactivation”, Department of Medicine III, Klinikum der Universität München, Munich, Germany; ^4^ Institute of Virology, School of Medicine, Technische Universität München & Helmholtz Zentrum München, Munich, Germany; ^5^ Department of Internal Medicine II, University Hospital Rechts der Isar, School of Medicine, Technische Universität München, Munich, Germany; ^6^ Institute of Clinical Chemistry and Pathobiochemistry, School of Medicine, Technische Universität München, Munich, Germany; ^7^ TranslaTUM, Center for Translational Cancer Research, Technische Universität München, Munich, Germany; ^8^ Institute of General Practice and Health Services Research & Institute for AI and Informatics in Medicine, School of Medicine, Technische Universität München, Munich, Germany; ^9^ Research Unit Gene Vectors, Helmholtz Zentrum München, Munich, Germany

**Keywords:** SARS-CoV-2, multiplex serology, COVID-19, coronavirus, nucleocapsid protein

## Abstract

Understanding immune responses to severe acute respiratory syndrome coronavirus 2 (SARS-CoV-2) is crucial to contain the COVID-19 pandemic. Using a multiplex approach, serum IgG responses against the whole SARS-CoV-2 proteome and the nucleocapsid proteins of endemic human coronaviruses (HCoVs) were measured in SARS-CoV-2-infected donors and healthy controls. COVID-19 severity strongly correlated with IgG responses against the nucleocapsid (N) of SARS-CoV-2 and possibly with the number of viral antigens targeted. Furthermore, a strong correlation between COVID-19 severity and serum responses against N of endemic alpha- but not betacoronaviruses was detected. This correlation was neither caused by cross-reactivity of antibodies, nor by a general boosting effect of SARS-CoV-2 infection on pre-existing humoral immunity. These findings raise the prospect of a potential disease progression marker for COVID-19 severity that allows for early stratification of infected individuals.

## Introduction

A novel betacoronavirus designated Severe Acute Respiratory Syndrome Coronavirus 2 (SARS-CoV-2) emerged in late 2019 and is responsible for the worldwide Coronavirus Disease 2019 (COVID-19) pandemic that has claimed almost six million human lives in two years (1, 2) (https://coronavirus.jhu.edu/map.html). SARS-CoV-2 infection is characterized by a wide diversity in clinical presentation, ranging from asymptomatic or mild disease to severe pneumonia and death (3). What causes/contributes to this remarkable diversity is not fully understood and has prompted investigations to identify predictors of disease progression (4).

Diagnosis of SARS-CoV-2 infection mostly relies on the detection of viral genomic material or viral antigens in nasopharyngeal or deeper respiratory specimens by PCR and rapid antigen tests, respectively (5–7). Unlike viral RNA and proteins, antibodies against SARS-CoV-2, once elicited, are detectable and persist beyond infection (8). Consequently, serological approaches have been developed with the objective of improving diagnostic sensitivity as well as determining infection rates more accurately. Besides, serological analyses have yielded valuable information crucial for understanding aspects relevant to host-virus interaction. These include duration, magnitude, and effectivity of the immune response, prediction of disease severity, estimation of vaccine efficacy and the need for boosting, identification of appropriate human donors for therapeutic convalescent serum, evaluation of immune evasion potential of emerging viral mutants, and identification of risk factors for Post-COVID-19 syndrome (6, 7, 9–11). Ultimately, accurate serological data are pivotal in informing effective and ethical response strategies to the COVID-19 pandemic.

Multiple serologic tests have been developed for COVID-19, utilizing a wide range of technologies including Enzyme-Linked Immunosorbent Assays (ELISA), chemiluminescent immunoassays (CLIA), lateral flow immunochromatographic assays (LFAs), luciferase immunoprecipitation system (LIPS), flow cytometry-based methods, and protein microarrays (6, 9, 12). High throughput assays, such as ELISAs, mostly rely on the detection of antibodies against SARS-CoV-2 spike (S) and/or nucleocapsid (N) proteins (6). Because assay sensitivity of existing commercial tests rarely exceeds 80% (13), several studies have investigated serological responses to additional viral proteins. Combinations of larger sets of viral antigens have been tested in a small number of studies using different approaches such as LIPS, antigen microarrays, and phage display (14–19). Although often limited to few serum samples, these studies already provided clear evidence that antigenicity of SARS-CoV-2 extends beyond S and N. However, partially inconsistent results on the antigenicity of some viral proteins were reported across these studies, perhaps owing to differences in the applied methodologies. In addition, cross-reactivity of antibodies against other coronaviruses circulating endemically within the human population may exist and limit specificity of serology-based approaches (6, 9, 20). Thus, obtaining clearer insight into breadth and magnitude of the humoral responses, how they differ in patients with diverse outcomes, and how past infections may influence responses to SARS-CoV-2, may require comprehensive serological profiling, preferably *via* multiple, complementary approaches.

For capturing the complete repertoire of antibodies generated after SARS-CoV-2 infection, we developed a multiplex assay covering the whole SARS-CoV-2 proteome and profiled immune responses in SARS-CoV-2 infected and uninfected cohorts. In addition, past infections with endemic alpha and beta coronaviruses were assessed by measuring antibody responses against the nucleoproteins of these viruses that are known to be highly immunogenic and abundantly expressed after infection. By correlating breadth, magnitude, and kinetics of the SARS-CoV-2-specific IgG antibody response as well as preexisting immunity to endemic coronaviruses with clinical course, we have identified candidate serological markers predictive of severe SARS-CoV-2 infection.

## Materials and methods

### Patients and sample collection

Human serum and plasma samples derived from the peripheral blood of SARS-CoV-2 infected as well as unexposed individuals were used in this study by permission of the Research Ethics Committee of the Technische Universität München (TUM; project no. 147/20, 476/20, and 639/21S) and the Ludwig-Maximilians-Universität München (LMU; project no. 17-455). Written informed consent was obtained from all donors. In total, 84 SARS-CoV-2-infected adults (age, 18 years to 85 years; mean age, 46.4 years; m:f, 50:34) that had been tested positive by RT-PCR (n=73) and/or by the approved, commercially-available, standardized serological assays (iFlash-SARS-CoV-2, Shenzhen Yhlo Biotech, and Elecsys, Roche) were included in this study. Serum was obtained by centrifugation of coagulated blood, obtained by venipuncture using serum vacutainers (Sarstedt). The cohort was classified into a) asymptomatic (7/84), b) symptomatic but not requiring hospitalization (36/84), c) requiring hospitalization but not intensive care (27/84), d) requiring intensive care (14/84). Two samples were available from each of 21 individuals and four samples from one patient. Also included in this study were 104 pre-pandemic healthy control sera collected in 2018 and 2019 (mean age and sex unknown), and sera of 30 healthy donors collected in 2020 (mean age, 42y, m:f 14:16), 15 of these as a follow-up sample to the previously mentioned pre-pandemic controls. All 30 healthy donors were negative for IgG antibodies against S and N of SARS-CoV-2. Sera from SARS-CoV-2-infected subjects and the healthy control group from 2020 were collected between February and December 2020. Serum and plasma samples were stored at -80°C until use. In **Figure 2** and **Figure 4**, the days after symptom onset are indicated for symptomatic donors. For asymptomatic donors, days since detection of infection is used as a proxy.

### Antigenic proteins

All 28 putative open reading frames of the SARS-CoV-2 prototype strain (Wuhan-Hu-1, MN908947.3) were recombinantly expressed as fusion proteins following transient transfection in HEK293T cells. A common expression plasmid (pcDNA3, Invitrogen) was used to clone codon-optimized, PCR-amplified, end-modified DNA constructs for the expression of coronavirus proteins as well as BFRF3 and EBNA1 of the Epstein-Barr virus strain B95.8 (GenBank: V01555.2), the human IgG1 constant region (GenBank: P01857.1) and GFP. By design of the expression plasmid, recombinant proteins were tagged C-terminally with six histidines (His_6_) that allowed for purification from transfected cell lysates using a 8M urea lysis buffer and Ni-NTA agarose affinity purification, as described previously (21). In brief, lysates of transfected cells were incubated overnight with Ni-NTA agarose beads (Qiagen). Subsequently, bound proteins were eluted using lysis buffer containing 0.5 M imidazole and eluted proteins detected using the anti-His_6_ mouse monoclonal IgG antibody 3D5. For expression of full-length S protein, the furin cleavage site was destroyed (R683A, R685A). In addition, S protein in native confirmation was purified in PBS from the supernatant of cells transfected with a C-terminally truncated version of S lacking the last 60 amino acids. Tetanus/diphtheria vaccine (Td-pur^®^ Astro Pharma) was diluted in 8 M urea buffer (21).

Quantity and size of all recombinant proteins were analyzed by Western blot using the anti-His_6_ mouse monoclonal IgG antibody 3D5. Concentrations of proteins were estimated by running aliquots on polyacrylamide gels. Following Coomassie dye staining, band intensities were compared with those of known concentrations of bovine serum albumin (BSA) that were run in parallel. All preparations were adjusted to approximately 10 µg/ml.

### Multiplex dot-blot assay and quantification of measurement

In order to detect serum IgG antibody responses to SARS-CoV-2 proteins, all samples were tested in a multiplex dot blot assay. Briefly, 5 µl of each concentration-adjusted recombinant protein was spotted on a nitrocellulose membrane. The membrane was then dried, blocked with 5% milk powder in PBS and co-incubated overnight at 4°C in a 3% milk buffer with an anti-His_6_ antibody (clone 3D5) and patient serum diluted 1: 500. The membranes were subsequently washed and incubated with fluorescence-labelled anti-mouse IgG antibody (LI-COR^®^ IRDye 680) and anti-human IgG antibody (LI-COR^®^ IRDye 800). The membranes were scanned in a LI-COR^®^ Odyssey FC scanner that reports results as arbitrary fluorescence units (AFU), returning a CW700 and a CW800 reading for each dot on the membrane corresponding to the protein concentration (anti-His_6_) and the human serum response respectively. A standard curve of recombinant His_6_-tagged human IgG as well as solvent (8M urea) and Ni-NTA agarose-affinity enriched mock-transfected HEK293T cell lysate were used for specific standardization and background correction, enabling blot to blot comparability.

### Data processing

Autofluorescence signals caused by the nitrocellulose membrane or the solvent as well as any possible fluorescence due to serum responses against HEK293T proteins were considered background and subtracted from readings for antigenic proteins. Background-subtracted AFU values for proteins in the CW800 and CW700 channels were converted to normalized arbitrary values using a simple linear regression model drawn from values obtained for serial dilutions of recombinant IgG for each individual membrane. Next, the quotient of normalized CW800 and CW700 values was formed to compensate for potential differences in the amount of sample protein spotted on the membrane. This normalized AFU value is used to describe sera IgG responses against viral proteins.

In order to identify positive antibody responses towards candidate antigens, a protein-specific cutoff was established for each of the SARS-CoV-2 proteins. The cutoff for each protein-specific response was arbitrarily defined as the mean plus two standard deviation value derived from identical dot blot assays performed using 104 pre-pandemic control sera. This approach correctly classified pre-pandemic control samples with a specificity of 95.2% - 100.0% (mean: 98.1%).

### Software and statistical methods

The distribution of quantitative data is presented by mean, range and standard deviation. Qualitative data is described by absolute and relative frequencies. Correlations between two parameters were evaluated using Spearman’s correlation coefficient (r). r values between 1.0 and 0.7, 0.7 and 0.5, 0.5 and 0.3, and below 0.3 were considered very strong, strong, moderate, and low correlations, respectively (22). Group differences were assessed by Mann-Whitney U tests. Hypothesis testing was performed at exploratory two-sided 5% significance levels. The cutoff values for calculating the odds ratios in **Figure 6C** were obtained *via* maximization of Youden’s J statistic (Youden index) in the receiver operating characteristic curves (ROC). Data was collected in Microsoft Excel sheets, analyzed using GraphPad Prism 8 and figures finalized in Adobe Illustrator CS.

## Results

### Multiplex analysis of the SARS-CoV-2-specific IgG response

**Figure 1:**
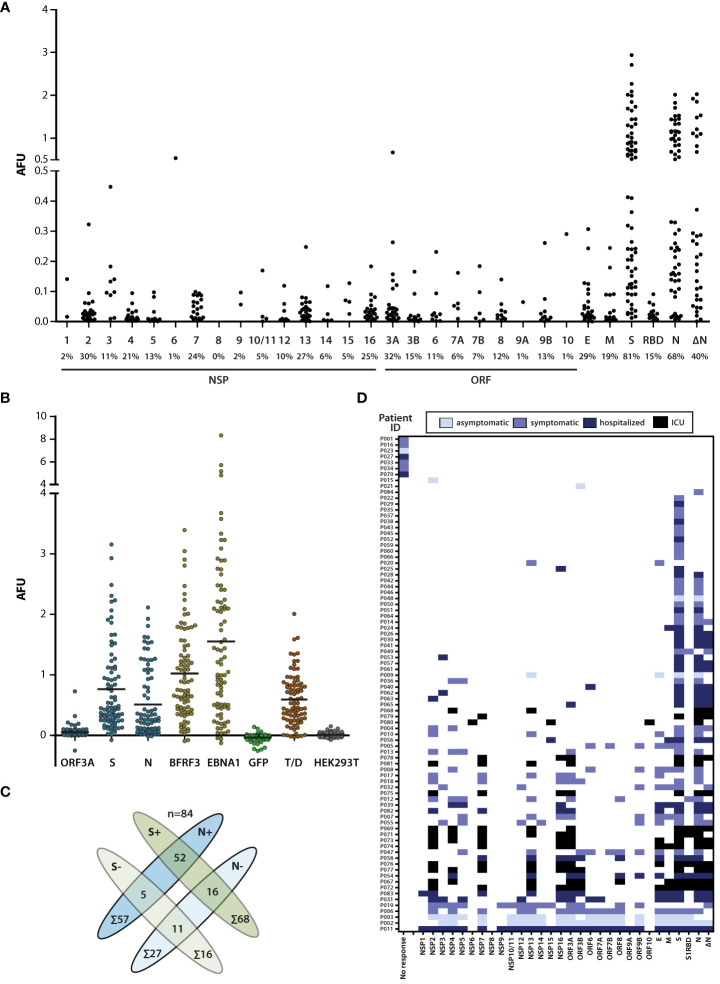
Broadness and magnitude of the SARS-CoV-2-specific IgG response. **(A)** Sera of SARS-CoV-2-infected donors (n=84) were probed for IgG responses against the SARS-CoV-2 proteome. IgG antibody responses are represented as arbitrary fluorescence units (AFU). The names of the viral proteins are depicted on the X-axis (NSP, non-structural protein; ORF, open reading frame) and the percentage of responding donors is indicated below the protein name. Only signal values above cutoff are shown. RBD, S protein receptor-binding domain; ΔN, nucleoprotein lacking aa 1-120. **(B)** Comparison of the magnitude of the antibody responses against ORF3A, S, N, and the major Epstein-Barr virus antigens BFRF3 (VCAp18) and EBNA1, as well as tetanus/diphtheria (T/D) antigens. GFP and HEK-Lysate purified over Nickel-NTA beads were used as controls. Total values are shown (no cutoff applied). Mean value of responses (horizontal lines): ORF3A: 0.054; S: 0.764; N: 0.510; BFRF3: 1.024; EBNA1: 1.553; GFP: -0.034; Tet/D: 0.593; HEK293T: 0.009. **(C)** Antibody responses against N and/or S protein. 68/84 donors showed responses against S and 57/84 against N. 52 donors had responses against both proteins, while 5 had responses against N and 16 responses against S only. **(D)** Individual antibody responses of SARS-CoV-2-infected donors with color-coded clinical grading. IgG responses against one or several viral antigens were detected in 77 SARS-CoV-2 infected donors while no virus-specific antibody responses above cutoff were detected in 7 donors.

All annotated open reading frames in the prototype SARS-CoV-2 strain Wuhan-Hu-1 coronavirus (MN908947.3) (2) were recombinantly expressed in human cells and purified proteins probed with sera from 84 SARS-CoV-2 infected donors with different degrees of disease severity. For better comparison with commercial tests frequently targeting the S1 receptor-binding domain (RBD) or a truncated version of N (ΔN), these antigen variants were also included. As depicted in **Figure 1A**, most proteins of the virus were targets of the humoral immune response. IgG responses against S and N were dominant, both in frequency and magnitude. Compared to full-length proteins, responses to the RBD and ΔN were lower, especially in the case of RBD, possibly due to its smaller size and the tendency of RBD-directed antibodies to be conformation-dependent (23). Responses against other viral proteins were detected in 0% (NSP8) to 32% (ORF3A) of all donors, albeit at titers that were approximately one order of magnitude lower compared to S and N (**Figure 1A**). For ranking these titers within a broader context, we assessed responses against tetanus/diphteria (T/D) vaccine antigens as well as two major antigens of the ubiquitous and persistent Epstein-Barr virus (EBV) (**Figure 1B**). Mean responses against S and N were in the range of those against T/D, and approximately twofold lower than those against EBNA1 and BFRF3 from EBV. IgG responses against ORF3a were on average 10-fold lower than those against the EBV antigens (**Figure 1B**).

S and N are the most commonly used targets in serological studies including diagnostics, so we assessed these responses further. S-specific IgG responses were detected in 81% (68/84) and N-specific responses in 68% (57/84) of the donors. 62% (52/84) of the donors had detectable responses against both antigens while 25% (21/84) had detectable responses against one of the two proteins only (**Figure 1C**). This finding that IgG responses against S and N do not completely overlap suggests that rates of sero-detection of infection could improve if both S and N are included as target antigens. The detection of positive responses against S and/or N in 87% of the donors by this multiplex assay (**Figure 1C**), and in 82% of the donors by a commercial ChemiLuminescent ImmunoAssay (iFlash, Yhlo) that contains N and S as antigens and enables detection of IgG and IgM responses (data not shown), verified suitability of this assay for studying antibody responses against SARS-CoV-2.

To address whether the sero-detection rate would improve when additional viral proteins were included as targets, and whether antibody responses correlated with disease severity, donors were subdivided into four groups by the presence of symptoms and the need of hospitaliation as described in the methods section (**Figure 1D**). Except for 7/84 of the donors in whom no IgG response was detected against any of the viral proteins, 92% (77/84) of the donors recognised at least one and frequently more than one viral antigen. Among those that recognized one or more viral antigens, 4/77 of the donors had no detectable response against S or N and would be identified as negative by currently available serum-based assays. Two of these donors were asymptomatically and two symptomatically infected (**Figure 1D**). These findings suggest that sensitivity of serological assays using viral antigens can increase but may not reach 100%, irrespective of the number of viral protein targets used. Although severely ill patients generally showed IgG responses against a broader set of ≥4 antigens, often including NSP2, NSP7, NSP13, NSP16 and ORF3A (**Figure 1D**), additional studies on larger cohorts are needed to substantiate this correlation.

### N-specific IgG responses correlate with disease severity

**Figure 2:**
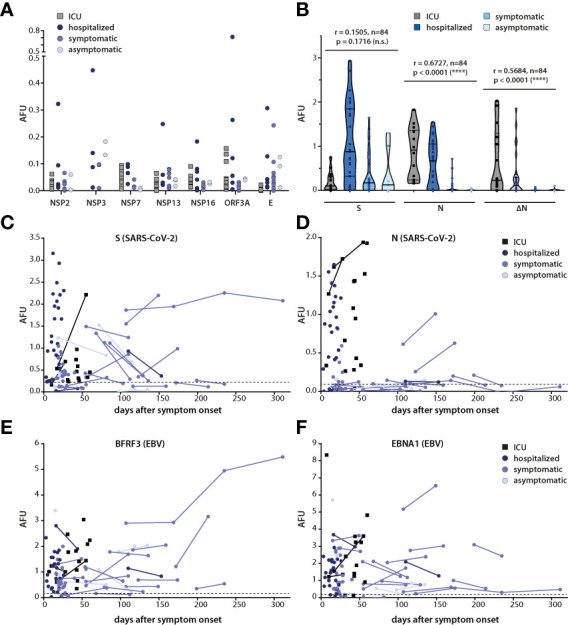
Correlation of kinetics and magnitude of the antibody response with disease severity. **(A)** IgG responses against selected SARS-CoV-2 antigens in the four clinical subgroups. **(B)** Correlation of IgG responses against S, N and ΔN with disease severity. Spearman correlation coefficient and p values are shown. **(C–F)** Antibody responses against S, N, BFRF3, and EBNA1 are displayed as arbitrary fluorescence units (AFU) in SARS-CoV-2-infected persons subdivided into the indicated four clinical subgroups. Lines connect multiple measurement points in the same individual. In **(A)** and **(B)**, only values above cutoff are shown, in **(C–F)**, total values are plotted, and protein-specific cutoffs are depicted as dashed lines.

The difference in the magnitude of the IgG response to different viral proteins between donors raised the question whether antibody titers correlated with severity of infection. Therefore, IgG responses in the four clinical subgroups were evaluated. Among the most common target antigens, responses against N and ΔN, but not those against S correlated with disease severity (**Figure 2B**). Responses against NSP7, NSP16 and ORF3a were also predominantly seen in subgroups with more severe course of the disease, but because of the small number of individuals for which IgG responses against these targets were detected, no firm conclusions can be drawn from these findings (**Figure 2A**).

To understand the kinetics of the responses, serum responses against S and N in the four cohorts were plotted relative to the time after infection (**Figures 2C, D**). Responses against BFRF3 and EBNA1 of the Epstein-Barr virus (EBV), which are generally detectable throughout life in most virus carriers, were included as controls (**Figures 2E, F**). Because only one of the donors in the SARS-CoV-2 cohort was EBV-negative, cutoffs for BFRF3 and EBNA1 were established using an EBV-negative control group (**Figures 2C–F**). The kinetics of the IgG response against S varied between donors. Titers appeared to decrease, increase or remain rather constant (**Figure 2C**) but no obvious association of these diverse patterns with disease severity was noted. Because too few donors with more than one measurement point showed responses against N, no conclusion can be drawn on the kinetic of the N-specific antibody response (**Figure 2D**). The magnitude of the antibody response against the EBV antigens did not correlate with severity of SARS-CoV-2 infection and titers generally remained similar over time (**Figures 2E, F**).

### Correlation of antibody responses against N of SARS-CoV-2 and endemic coronaviruses

Although antibody responses against N correlated with disease severity (**Figure 2B**), the utility of N as a clinical marker has been questioned due to concerns of false-positive sero-detection rates (24). While responses against ΔN also correlated with disease severity, seropositivity dropped from 68% to 40% when ΔN instead of N was used (**Figure 1**), curbing its clinical utility.

**Figure 3:**
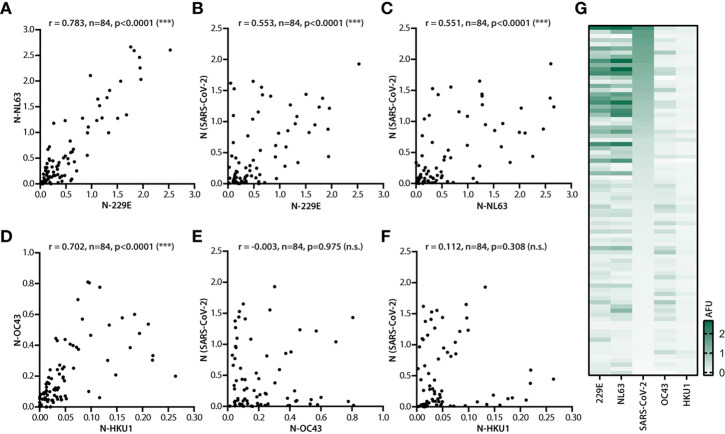
Correlation of IgG responses against N of different coronaviruses. Serum samples of 84 donors (n=84) were analyzed for IgG responses against N of SARS-CoV-2 and N of endemic coronaviruses 229E, NL63, OC43 and HKU1. **(A)** Correlation analysis of the antibody response against N of alphacoronaviruses NL63 and 229E. Correlation of the antibody response against N of SARS-CoV-2 and 229E **(B)**, as well as SARS-CoV-2 and NL63 **(C)**. Correlation analysis of the antibody response against N of betacoronaviruses OC43 and HKU1 **(D)**, N of SARS-CoV-2 and OC43 **(E)**, and N of SARS-CoV-2 and HKU1 **(F)**. Spearman correlation coefficient and p values are displayed. **(G)** Heat map analysis of the antibody response against N of 229E, NL63, SARS-CoV-2, OC43 and HKU1 in SARS-CoV-2 infected donors (n=84). The strength of the response was measured in AFU without applying cutoff and is depicted in green. Donors were sorted by decreasing titers against N of SARS-CoV-2. ns, not significant; ***, p<0.0001.

SARS-CoV-2 shares amino acid sequences and antigenic T and B cell epitopes with the endemic human coronaviruses (HCoVs) 229E, OC43, NL63 and HKU1, that cause common cold, as well as with MERS-CoV and SARS-CoV that both can cause severe and fatal respiratory disease (9, 25). Among these coronaviruses, population level seropositivity for MERS-CoV and SARS-CoV is low, and therefore as far as cross-reactivity to SARS-CoV-2 goes, the endemic HCoVs are of foremost concern (9). Therefore, IgG responses against N of the endemic coronaviruses were measured in the study cohort and correlated with responses to each other as well as to N of SARS-CoV-2 (**Figure 3**).

In donor sera with measurable IgG responses, the magnitudes of the responses against N of the two alphacoronaviruses NL63 and 229E (r = 0.783), and the two betacoronaviruses OC43 and HKU1 (r = 0.702) correlated very strongly (**Figures 3A, D**). The amino acid sequences of N of the two alphacoronaviruses and the two betacoronaviruses are conserved to 46% and 64%, respectively. This conservation provides a plausible explanation for cross-reactivity and, therefore, for the observed correlation between antibody signals against N of viruses of the same genera. No correlations were detected for IgG responses against N of viruses of different genera (NL63 vs. OC43: r = -0.001; 229E vs>. OC43: r = 0.071; NL63 vs. HKU1: r = -0.021; 229E vs. HKU1: r = -0.003). Despite the observed correlation within a genus of the endemic coronavirus strains, no correlation between responses to N of endemic betacoronaviruses and N of SARS-CoV-2, also assigned to the *Betacoronavirus* genus, was noted (**Figures 3E, F**). Rather surprisingly, analyses returned much higher and significant correlation coefficients for responses against N of SARS-CoV-2 versus those against N of endemic alphacoronaviruses (**Figures 3B, C**), even though the N amino acid sequence identities between SARS-CoV-2 and the two betacoronaviruses OC43 and HKU1 (34.6% and 33.9% respectively) are higher than those between SARS-CoV-2 and the two alphacoronaviruses NL63 and 229E (32.6% and 28.4%, respectively) (9).

A side by side comparison of the IgG signals revealed that in spite of the overall higher correlation between N of SARS-CoV-2 and the endemic alphacoronaviruses, responses to alphacoronavirus N and SARS-CoV-2 N did not fully overlap across donors (**Figure 3G**). This was in contrast to the much more consistent visible overlap between responses against N of 229E and N of NL63 or OC43 and HKU1. These findings further substantiated the notion of cross-reactivity of IgG responses against N of endemic alphacoronaviruses or betacoronaviruses, but indicated that cross-reactivity does not fully explain the correlation between IgG responses to N of SARS-CoV-2 and the endemic alphacoronaviruses.

### Dynamics of IgG responses against endemic coronavirus N and association with severity of infection

A possible explanation for the observed concomitant increase in antibody titers (**Figure 3G**) was a boosting effect of SARS-CoV-2 infection on pre-existing humoral immune responses against N of alphacoronaviruses. In order to address whether antibody titers vary over time, we performed assays using sera from later time points where available. For comparison, the antibody response in sera of healthy controls collected before the pandemic (HC 2018/19) was assessed in parallel. Because endemic coronavirus infections are generally seasonal and peak in the northern hemisphere during winter months (26), we also included healthy controls that had been recruited during the winter months of the pandemic (HC 2020). From some of these donors, serum samples were available from 2018/2019, allowing us to study the progression of the antibody responses over time in a non-SARS-CoV-2 infection background.

**Figure 4:**
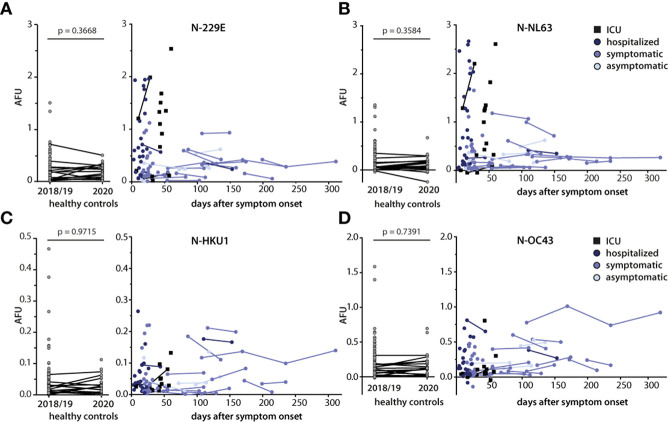
Kinetics of IgG responses against N of endemic coronaviruses and association with disease severity. Antibody responses against N of endemic coronaviruses 229E **(A)**, NL63 **(B)**, HKU1 **(C)**, and OC43 **(D)** were measured in sera of SARS-CoV-2 infected donors (n=84, total time points n=108), in sera of healthy controls collected before the pandemic (2018/2019, n=104), and in sera of a partly overlapping set of healthy controls (n=30) collected during the pandemic (2020, left graphs). SARS-CoV-2 infected donors were grouped according to disease severity. Results are plotted relative to the time of symptom onset (day 0). Individual lines connect serial measurements in the same individual. Mann-Whitney *U* test was used to compare IgG responses in the two healthy control groups.

Overall N-specific antibody responses against the alphacoronaviruses 229E and NL63 (**Figures 4A, B**) were found to be higher in magnitude compared to those against the betacoronaviruses HKU1 and OC43 (**Figures 4C, D**). For the four HCoVs, no assays are available to identify virus-naïve versus infected individuals. Therefore, no cutoffs could be established for antibody responses against these viruses. However, when the SARS-CoV-2 N-specific cutoff was applied as a proxy, the positivity rate for IgG response to the alphacoronaviruses was higher in the SARS-CoV-2-infected cohort compared to the SARS-CoV-2 negative samples. Moreover, within the SARS-CoV-2-positive cohort, seropositivity rates as well as the magnitudes of the responses were higher against alphacoronaviruses compared to the betacoronaviruses. This was especially the case in more severely ill subgroups of the cohort (**Figures 4A, B**). Unfortunately, we had no access to sera of these patients from later time points. However, in those cases where serum samples from multiple time points were available, signals against the different N remained stable up to 10 months after SARS-CoV-2 infection and up to two years in healthy donors. Furthermore, no appreciable changes in the serum response against the different N were detected in serum samples from a symptomatically infected donor collected 1.5 years before and at four time points within one year post SARS-CoV-2 infection (data not shown). Taken together, these findings indicate that SARS-CoV-2 infection does not lead to lasting alterations in the humoral immunity including responses to endemic coronaviruses.

### Antibodies against N of SARS-CoV-2 and N of alphacoronaviruses do not cross-react

**Figure 5:**
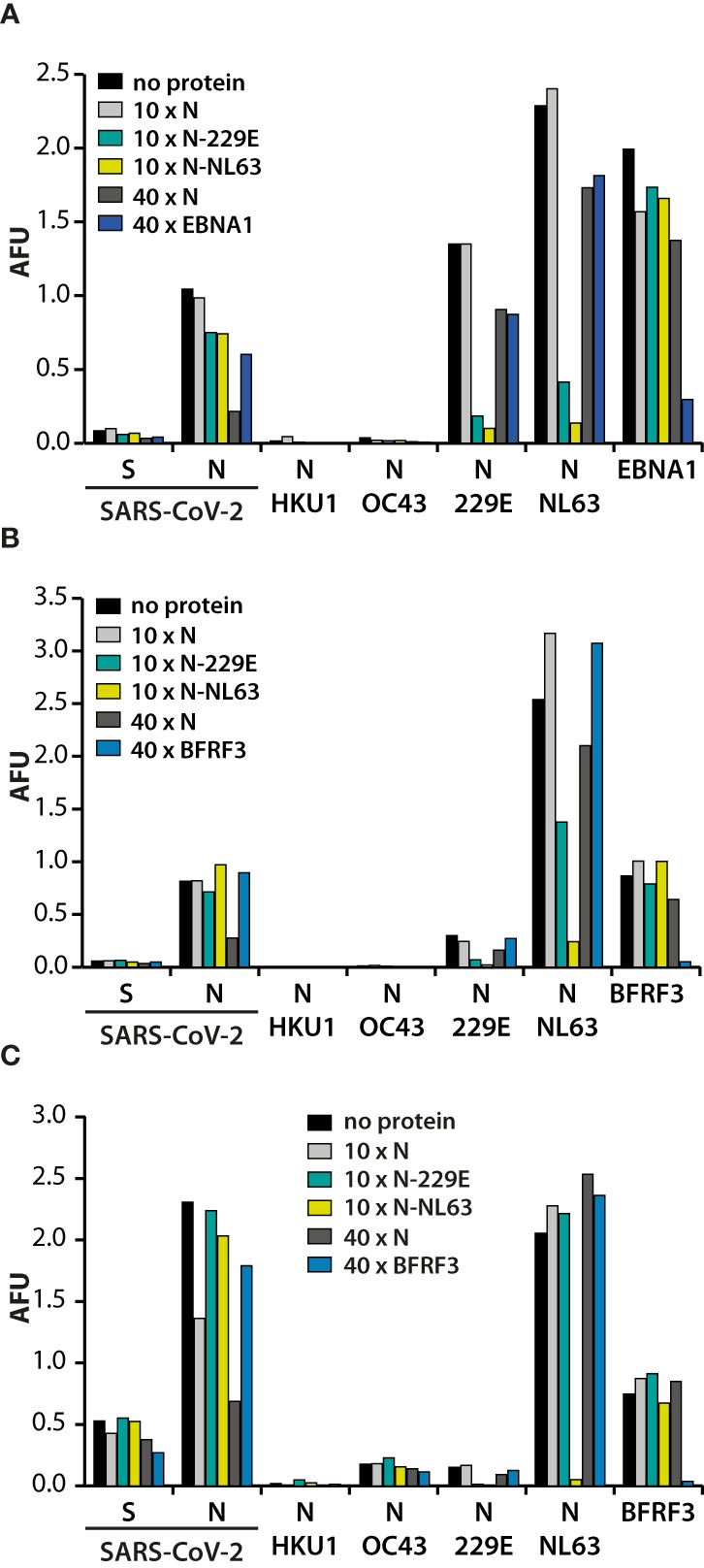
Specificity of the antibody responses against N of SARS-CoV-2 and the alphacoronaviruses. Serum samples of three SARS-CoV-2 infected donors **(A–C)** were either left untreated (no protein), or supplemented with the indicated proteins in a 10- or 40-fold excess of the amount spotted on the membranes and then probed against S and N of SARS-CoV-2, as well as the N proteins of the endemic HCoVs. Antibody responses against BFRF3 and EBNA1 were included as controls, depending on the previously established, individual response patterns.

Besides anamnestic immune reactivation, the overall correlation observed between responses to N of SARS-CoV-2 and N of alphacoronaviruses could also have been caused by antibody cross-reactivity (**Figure 3**). In order to address this issue, sera recognizing N of SARS-CoV-2 and N of alphacoronaviruses were preincubated for one hour with a 10- or 40-fold excess of an N and then used in the multiplex assay (**Figure 5**). A 10-fold excess of N of SARS-CoV-2 hardly influenced serodetection of N of SARS-CoV-2 whereas a 40-fold excess specifically lowered the signal by more than half. Preincubation with N of SARS-CoV-2, either at 10-fold or 40-fold excess barely affected IgG signals to N of NL63 or to N of 229E. This indicated that IgG responses to N of alphacoronaviruses were distinct from those to N of SARS-CoV-2. Moreover, neither a 10-fold excess of N of NL63 that almost completely abolished recognition of this antigen, nor a 10-fold excess of N of 229E that strongly reduced its serodetection, had an appreciable effect on the recognition of N of SARS-CoV-2 (**Figures 5A–C**). In addition, serodetection of N of SARS-CoV-2, but not of the alphacoronaviruses, was diminished in buffers containing ≥4 M urea (data not shown), indicating that antibodies against N of SARS-CoV-2 were of lower avidity, as reported before (27). These findings further substantiated the notion that the IgG responses that had been mounted against N of SARS-CoV-2 were not crossreactive against N of alphacoronaviruses and vice versa.

Where untreated sera demonstrated responses to both alphacoronaviruses, pre-incubation with a 10-fold excess of N of either alphacoronavirus usually led to diminished recognition of both, demonstrating that antibodies against N of 229E and NL63 are cross-reactive. However, in some donors high responses against N of NL63 and much lower responses against N of 229E were detected (**Figures 5B, C**). In these cases, preincubation with N of 229E had moderate effects on serodetection of N of NL63, demonstrating that in these cases antibody responses were strain-specific.

In contrast to N of the alphacoronaviruses and the EBV proteins that were included as controls, the high concentration of N required to diminish recognition indicated that the antibody response was of high titer but low affinity. Taken together these findings suggested that SARS-CoV-2 infection induced the production of novel antibodies recognizing SARS-CoV-2-N, but not N of alphacoronaviruses.

### IgG responses against N of the alphacoronaviruses correlate with disease severity

**Figure 6:**
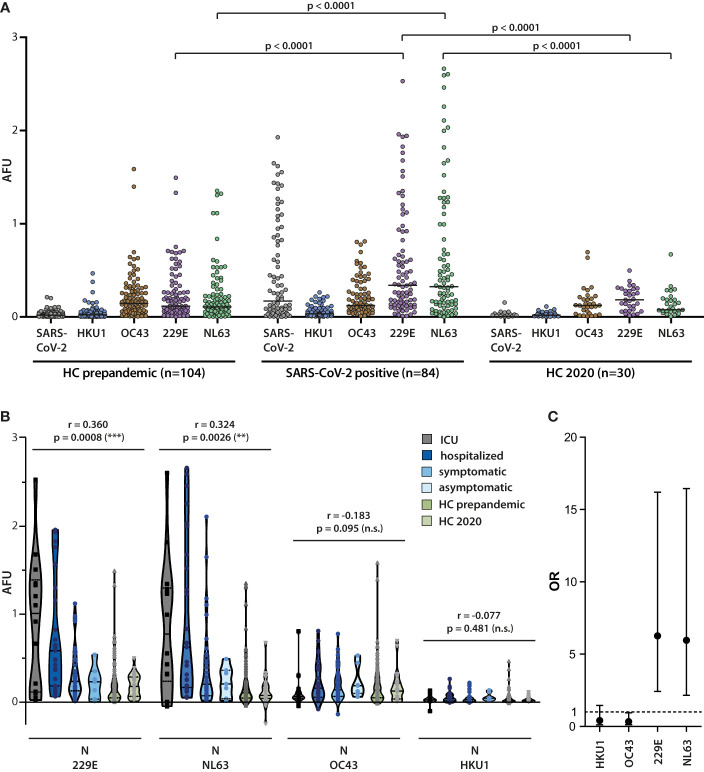
Correlation of alphacoronavirus N-specific IgG responses with COVID-19 severity. **(A)** The magnitude of the IgG antibody response against N of SARS-CoV-2 and the endemic coronaviruses HKU1, OC43, 229E, and OC43 was measured in sera from SARS-CoV-2 infected donors (n=84) and healthy controls (n=104) collected in 2018/2019 (HC pre-pandemic) as well as from healthy controls (n=30) collected in 2020. Mean values are indicated by horizontal lines. Results of Mann-Whitney *U* test are depicted. **(B)** Correlation of antibody responses with disease severity. Antibody responses against N of the indicated endemic coronaviruses were analyzed in the four indicated patient cohorts and healthy controls. Spearman correlation coefficient and p values are displayed. **(C)** Odds ratios (OR) for the association of serum responses to individual alpha- and betacoronavirus N proteins with hospitalized versus non-hospitalized infection events are shown along with the 95% confidence intervals. The serum response cutoff values used for calculating the OR were determined by Youden-Index maximization on ROC curves for individual N-specific IgG responses. Sensitivities (se) and specificities (sp) for the applied cutoffs were as follows: 229E se 68%, sp 74%; NL63 se 54%, sp 84%; OC43 se 63%, sp 16%; HKU1 se 10%, sp 79%.

In a side-by-side comparison, a significantly higher antibody titer against N of the alphacoronaviruses, but not betacoronaviruses became apparent in the SARS-CoV-2 infected cohort (**Figure 6A**). Moreover, mean antibody titers against N of 229E and NL63, but not OC43 or HKU1, correlated positively with disease severity, while mean responses in healthy controls were similar as in the asymptomatically SARS-CoV-2 infected (**Figure 6B**). To assess the strength of this association, ROC-analyses were performed to establish cutoff values that would help in differentiating between high and low titer responses in non-hospitalized (“asymptomatic” and “symptomatic”) and hospitalized (“hospitalized” and “ICU”) subgroups. ROC-analyses were performed using Youden’s J statistic (Youden index) in order to maximize both sensitivity and specificity. Odds ratios calculated with these benchmarks are depicted in **Figure 6C** and revealed that the odds of measuring a titer above the defined benchmark for N of 229E or NL63 were higher in the hospitalized group than in the non-hospitalized group.

## Discussion

Based on former serological studies of SARS-CoV and MERS, most assays measuring humoral responses against SARS-CoV-2 target S and N as antigens (9, 25). In the present study, we assessed breadth and magnitude of the IgG responses against the whole proteome of SARS-CoV-2 by probing convalescent sera from 84 individuals, ranging in infection severity from asymptomatic to severe illness, against full length recombinant viral proteins expressed in human cells. Our results show that S and N are immunodominant but not exclusive targets of the antibody response. Serum antibodies targeted a broad set of viral proteins, although titers were approximately one order of magnitude lower for other proteins compared to S and N. These differences in titers may pose challenges to assay development, account for some of the reported inconsistencies in serological responses, and emphasize the importance of consolidating findings using multiple approaches (17, 19).

The number of viral antigens targeted by serum antibodies ranged from 0 to 25 and encompassed all viral proteins except NSP8. Responses against S, N, and nonstructural antigens such as NSP2 and NSP12 overlapped largely, but not completely, suggesting that a combination of multiple antigens in serological tests may increase sensitivity and reliability of seroprevalence studies.

Mean responses against S exceeded those against N, except for ICU patients. This contrasts with a number of studies demonstrating correlations between IgG-S1, S1-RBD, and neutralizing antibody levels and disease severity (28, 29). However, our results are in line with other studies showing that in individuals with severe COVID-19, N-specific antibody titers prevail (30, 31). In our study cohort, IgG responses against full-length N correlated with disease severity in the majority of patients. This accords with a previous report in which antibodies against an epitope in N that were detected in 27% of patients, were found to predict COVID-19 severity (32). Such predictive value, along with early detectability following infection as well as broad and high titer responses, render N a promising candidate for prognostic serological assays (33). However, clinical utility could be hampered by its potential cross-reactivity with endemic HCoVs (24, 25), which are highly prevalent in the human population worldwide with seropositivity reported to approach 90% except for the less common HKU1 strain (25, 34–37). Former epitope profiling experiments have described shared epitopes between SARS-CoV-2 and the betacoronavirus OC43, but to our knowledge, no shared epitopes between SARS-CoV-2 and the alphacoronaviruses have been identified (25, 38, 39). Our findings show that antibody responses against N of SARS-CoV-2 strongly correlate with N of alphacoronaviruses, but not with N of betacoronaviruses, a rather surprising finding given N of SARS-CoV-2 shares a higher degree of homology with the latter.

Mean age differences between the healthy controls (42 years) and the SARS-CoV-2 cohort (46 years) were excluded as possible confounding factor because antibody titers against HCoVs remain constant over time or even decrease with age (25, 34, 38).

Despite the small sample size, we found detectable responses to N of alphacoronaviruses in most of our donors, thereby reflecting the endemic nature of these viruses. Pretreatment of serum reactive to N of both alphacoronaviruses with a tenfold excess of either N almost completely abrogated these signals. If, however, only N of NL63 was recognized, the serum signal was strongly reduced when N of NL63, but not or only partially when N of 229E was added. These results demonstrated that some but not all antibody responses against N of alphacoronaviruses are cross-reactive. By contrast, high titer responses against N of alphacoronaviruses were hardly affected by pre-treatment of reactive serum with N of SARS-CoV-2 and high titer responses to N of SARS-CoV-2 were unaffected by pre-treatment of reactive serum with N of alphacoronaviruses. Thus, in donors were reactivity to N of SARS-CoV-2 as well as N of alphacoronaviruses was observed, these were probably caused by independent components of humoral immunity.

While our results argued against cross-reactivity of the antibody response, high titer responses against N of alphacoronaviruses were detected especially at rather early time points, raising the possibility that SARS-CoV-2 infection boosted pre-existing immunity. Titers to endemic coronavirus N or to control antigens EBNA1, BFRF3, and T/D did not change appreciably over time, making a general boosting effect on humoral immunity after SARS-CoV-2 infection unlikely. Moreover, HCoVs-specific antibody titers did not change remarkably over time in samples from healthy donors collected before and during the pandemic and in follow-up samples available from some of the donors, excluding major seasonal outbreaks during the observational period that might have led to increased responses against any of the HCoVs in the SARS-CoV-2 cohort and to a skewing of our results. Although we cannot formally exclude surges in titers soon after infection followed by a rapid decline, this scenario appears unlikely for two reasons. First, such an effect would be expected not to be exclusive for alphacoronaviruses and second, the half-life of IgG in serum is about 30 days (40). Nevertheless, a boost of heterologous humoral immunity cannot be ruled out and warrants further studies involving serum samples taken from the same person before and after SARS-CoV-2 infection.

Regardless of the underlying mechanism, our findings demonstrate that humoral responses to N of SARS-CoV-2 and alphacoronaviruses are tightly linked. Given the important clinical implication, such an association between HCoV infection and COVID-19 disease has been addressed in several studies. Contrary to our results, most of these studies failed to detect associations, which may be explained by differences in study design and/or implementation. For example, Anderson et al. compared antibody responses against HCoVs and SARS-CoV-2 in individuals before and after SARS-CoV-2 infection and showed that antibodies against HCoVs are boosted by, but do not confer cross-protection against SARS-CoV-2 infection or hospitalization (38). Unfortunately, antibodies against N of the alphacoronaviruses were neither measured pre/post infection, nor correlated with disease severity. Likewise, a study in children found no evidence of cross-protective immunity linked to previous infection with seasonal HCoVs as prevalence of HCoVs infections were similar in children with SARS-CoV-2 infection, children with multisystem inflammatory syndrome (MIS), and SARS-CoV-2 negative controls. However, children infected with SARS-CoV-2 often develop no, or only mild symptoms and such patients also failed to show increased levels of responses against N of HCoVs in our study. Interestingly, MIS patients not only showed increased titers of SARS-CoV-2-specific antibodies but also a significantly increased prevalence of specific antibodies to N of HCoV, a finding the authors interpreted as anamnestic immune response, without entertaining alternative possibilities (41). In a recent longitudinal study, antibody levels against HCoV N were analyzed in 33 study participants from which serum samples pre and post SARS-CoV-2 infection were available (42). Although a general upward trend in antibody titers against N of HCoVs was observed, overall an inverse relationship between anti-HCoV and SARS-CoV-2-specific antibody titers was detected. However, the study cohort consisted of asymptomatically or mildly infected health care workers and did not include severely ill patients (42). Using a semi-quantitative approach, two studies measured antibodies against N of HCoVs with a recently marketed line-immunoassay and detected lower levels of antibodies against OC43 in patients with critical disease compared to other COVID-19 inpatients (43), and a correlation of antibodies against the alphacoronaviruses with clinical severity score (44). Recently, Wratil et al. corroborated these findings and reported elevated antibody titers against endemic alphacoronaviruses especially in male and critically ill COVID-19 patients (45). However, cross-reactivity of antibodies and boosting effects on preexisting immunity could not be excluded in these studies. By ruling out these possibilities, our results argue against the concept of “original antigenic sin” and antibody-dependent enhancement of SARS-CoV-2 infection that had been proposed previously (45). How then increased antibody titers against N of alphacoronaviruses affect COVID-19 disease severity still remains to be determined. The association could be merely circumstantial and a sheer manifestation of an impaired immune response against coronavirus infections in general, as described for patients with autoantibodies to type I interferons (46). However, the unexpected association with alphacoronaviruses but not betacoronaviruses argues against subclinical immunodeficiency. Further insight may be gained by answering the question whether an increased risk for severe COVID-19 is associated with infection of either, or a specific alphacoronavirus. Due to the tight correlation in N-specific antibody responses, we were unable to discriminate between 229E and NL63 infections in most patients. If infection with either strain increases the risk of severe COVID-19, host immune responses may play a critical role, as implied before (47). Conversely, an association with specifically one of the strains, despite the overall high degree of homology, might implicate differences in pathobiology. Of note, the two viral strains engage different host cell receptors and possibly differ in cell tropism (48). Future investigations addressing similarities and differences between the viral family members are expected to provide a deeper understanding of the biological mechanisms underlying variable severity of COVID-19, and provide novel insights into how past infections with rather low-pathogenic viruses may impact on patient outcome and how they relate to known risk factors such as advanced age, sex, ethnicity, socioeconomic status, and comorbidities (49).

## Data availability statement

The raw data supporting the conclusions of this article will be made available by the authors, without undue reservation.

## Ethics statement

The studies involving human participants were reviewed and approved by Research Ethics Committees of the Technische Universität München and Ludwig-Maximilians-Universität München. The patients/participants provided their written informed consent to participate in this study.

## Author contributions

UB and JM designed the study and analyzed the data. AWM, MF, LW, JE, JS, MQ, AM, and UP recruited participants and collected samples in the clinic. HM, CW, and JR processed and biobanked the samples. CW, JR, JE, JS, UP, and EP managed the clinical data. WH provided antigen expression constructs. JN, EP, KD, and JM produced the antigens and performed serological assays. AH gave support in statistical analysis. JN, EP, MF, CW, JE, and AM contributed to critical interpretation of the results. JM, JN, and EP wrote the manuscript and all authors approved the final version as submitted to the journal.

## Funding

Funding was received by Wilhelm Sander-Stiftung (to AM, project no. 2018.135.1). The study was supported by the Project “Virological and immunological determinants of COVID-19 pathogenesis – lessons to get prepared for future pandemics (KA1-Co-02 “COVIPA”)”, a grant from the Helmholtz Association's Initiative and Networking Fund (to UP), and by the BMBF initiative NaFoUniMedCovid19 (01KX2021), subprojects B-FAST/PREPARED (to UP).

## Acknowledgments

Excellent technical assistance by Grit Müller-Neumann and Dorothea Seubert is greatly appreciated. We are indebted to Dinesh Adhikary for his invaluable IT support and critical reading of the manuscript.

## Conflict of interest

Author UP is receiving grants from Hoehnle AG, SCG Cell Therapy and VirBio and personal fees from Abbott, Abbvie, Arbutus, Gilead, GSK, J&J, Roche, Sanofi, Sobi and Vaccitech. UP is co-founder and shareholder of SCG Cell Therapy.

The remaining authors declare that the research was conducted in the absence of any commercial or financial relationships that could be construed as a potential conflict of interest.

## Publisher’s note

All claims expressed in this article are solely those of the authors and do not necessarily represent those of their affiliated organizations, or those of the publisher, the editors and the reviewers. Any product that may be evaluated in this article, or claim that may be made by its manufacturer, is not guaranteed or endorsed by the publisher.
